# Scabies among Dutch higher education students: do cases notify their contacts and do contacts take adequate measures?

**DOI:** 10.1186/s13071-026-07301-8

**Published:** 2026-03-16

**Authors:** Wilma A. Stolk, Marloes D. Stradmeijer, Hélène A. C. M. Voeten, Inge M. Lewis-van Disseldorp, Diane de Zwart-Slats, Fraukje E. F. Mevissen

**Affiliations:** 1 Department of Infectious Diseases, Public Health Service (GGD) of Rotterdam-Rijnmond, Rotterdam, the Netherlands; 2 Department of Public Health, Erasmus MC, University Medical Center Rotterdam, Rotterdam, the Netherlands; 3 Public Health Service (GGD) Hecht Hollands Midden, Leiden, the Netherlands

**Keywords:** Scabies, Young adults, Students, Contact notification, Preventive treatment, Behaviour, Questionnaire

## Abstract

**Background:**

Scabies incidence has rapidly increased in the Netherlands, particularly among higher education students. We hypothesized that effective control in this group is hindered by poor contact notification by indexes and limited treatment by contacts. We assessed this in a questionnaire study.

**Methods:**

An online questionnaire was distributed to students, focusing on their adherence to notification (indexes), and treatment and hygiene recommendations (contacts), as well as their health seeking behaviour.

**Results:**

Of the indexes (*n* = 308), 59% had experienced multiple scabies episodes and almost two-thirds had not been notified beforehand. Notably, 48% self-diagnosed their condition. Most indexes notified all regular (bed) partners and housemates, 53% also notified all casual bed partners. Contacts (*n* = 269) were usually notified by the index; in 57% of cases the notifier was a housemate. After notification, many contacts washed their bedding (85%), washed their clothes or put them in bags (81%); applied permethrin/benzoate cream (78%), avoided physical contact with others (74%); many also notified their own contacts (59%). The general practitioner was frequently contacted, especially by indexes, although experiences were often rated poorly owing to conflicting or incorrect information.

**Conclusions:**

The high proportion of unnotified indexes suggests a notification gap. Especially casual bedpartners often remain unnotified, presenting a risk for onward transmission. Contacts generally took adequate measures, although premature notification of their contacts may lead to unnecessary treatment or ‘notification fatigue.’ Enhancing healthcare support and communication might help to improve notification and prophylactic treatment behaviours and may play a crucial role in breaking the chain of transmission.

**Graphical Abstract:**

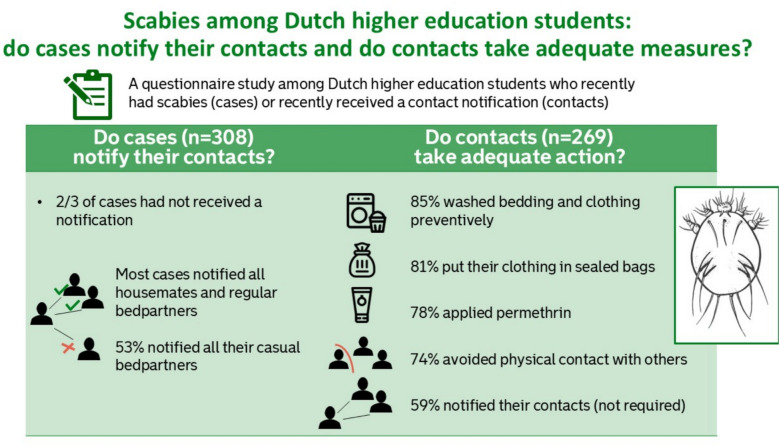

## Background

Scabies is a contagious skin condition, caused by the scabies mite *Sarcoptes scabiei* (var. *hominis*) and associated with intense itching, especially at night. The infestation is directly transmitted through prolonged skin-to-skin contact. Indirect transmission can also occur via fomites (e.g. via shared mattresses, bed linen, clothing, chairs/couches), although this is deemed uncommon in ordinary scabies [1]. The infestation has a long incubation time, as signs and symptoms relate to the host’s immune response to the mite that typically appears 4–6 weeks after the first contagion. During this incubation period, people can pass on the infestation to others. Scabies can be treated with permethrin or benzyl benzoate cream or oral ivermectin [2]. A second treatment after 7–14 days is recommended to kill newly hatched mites [2]. To prevent re-infestation, potentially infected household members and other close contacts should be treated simultaneously with the index and environmental decontamination measures should be applied (e.g., washing of clothing, bed linen, towels and vacuum cleaning of carpet, couch) [2]. The many steps involved make scabies treatment cumbersome and failure-prone [3, 4].

The scabies incidence in the Netherlands has risen sharply in the past decade, with more general practitioner (GP) consultations [5], scabicide dispensations [5] and institutional outbreaks (e.g., in nursing homes or daycare centres). This resulted in increased pressure on GPs [5], dermatologists and public health services. Similar increases are seen in other European countries [6]. The increase in scabies incidence is not fully understood. Potential contributing factors include increased importation (e.g. through tourism/displaced populations), changes in social behaviour, and altered living conditions [6]. Reduced mite susceptibility to treatment may also contribute [2, 7], but is difficult to separate from other causes of treatment failure such as suboptimal treatment application or incomplete contact treatment [8]. Changes in scabies health seeking behaviour could also contribute to the increase.

GP data show that the incidence is particularly high among older adolescents and young adults [5]. Based on stories from non-scientific media, scabies seems to be particularly problematic among higher education students, with frequent outbreaks in student houses (e.g., [9–11]). Students constitute a specific risk group for scabies owing to their extensive contact networks, shared housing facilities and behavioural factors (e.g. sharing of bedding, clothes). Therefore, specific attention to students is needed in the prevention and control of scabies in the Netherlands.

Scabies management practices need to be strengthened to stop the epidemic. National guidelines for scabies treatment stress the importance of treating potentially infected contacts at the same time as the index [12], but this is often difficult in practice. It requires indexes to notify all their contacts (e.g., housemates, sexual partners, guests, other contacts), who must then follow the recommended hygiene and treatment guidelines. Failure to treat all contacts has been associated with increased treatment failure [3]. Research on contact notification for sexually transmitted diseases and coronavirus disease-19 (Covid-19) shows this is often challenging, due to societal dynamics, disease characteristics or stigma [13, 14]. We hypothesize that poor contact treatment hinders scabies control among students. Because of the long incubation time, cases need to trace back all their contacts from 6 weeks before the start of symptoms till present. Cases may prematurely conclude that contacts, who remained symptom-free, have not contracted the infestation and may avoid informing them. Similarly, contacts may prematurely conclude that they are fine and may therefore refrain from taking preventive measures.

To inform interventions for scabies control, we assessed whether students with scabies notify all their contacts (housemates, sexual contacts, guests, others), and whether notified students take adequate measures. Health seeking behaviour and reasons for non-compliance with recommended measures were also evaluated.

## Methods

### Study design and study population

This study comprised an online cross-sectional questionnaire, among higher education students in the Netherlands aged 18–30 years, who had experienced scabies or had been in contact with someone with scabies in the 12 months prior to the survey. Higher education students are students who were enrolled in Dutch higher vocational education (in Dutch: Hoger Beroepsonderwijs, HBO) or universities [15]. In the Netherlands, respectively 417,000 and 337,000 students were enrolled in HBO institutions and universities in the study year 2022–2023. About 39% of HBO students and 71% of university students live away from their parents, e.g. in a rented private room in a student house or student housing complex, often sharing facilities such as kitchen, bathroom, toilet and living room, with one or more other students/persons.

### Recruitment

Participants were recruited via higher education institutions, local study and student associations, student GPs and public health services in Rotterdam and Leiden. For a wider reach, we used snowballing and also approached national student associations and public health services in other student cities. A combination of online and offline methods were used. Digital leaflets were distributed via online education platforms or mailing list of consenting higher education institutions, study and student associations. Physical posters and flyers were left at student notification boards/flyer holders, at student GPs and at public health services. In Rotterdam, post mailings were sent to all addresses in selected major student housing complexes. Students were provided with a QR code or direct link to the questionnaire. The online questionnaire was open for responses from 12 May to 24 July 2023.

### Questionnaire

On the online questionnaire’s opening page, we provided clear, non-technical information about the study’s purpose, key characteristics (voluntary participation, anonymous data collection, 10–15 min survey completion time) and eligibility criteria (see definition study population). We provided a weblink and email address for more detailed information. To encourage participation, we offered a chance to win one of five €50-gift vouchers. We then asked for digital informed consent.

Participants providing informed consent were led to the main questionnaire. The introductory section started with questions about demographics and basic scabies knowledge. We then asked whether and when participants had had scabies (indexes) or had been in contact with someone with scabies (contacts). For any student without such experience the questionnaire ended here, and these respondents are not considered in the current analysis. Respondents then continued with either the ‘index’ or ‘contact’ questionnaire, depending on their most recent experience. If their most recent experience had been both as index and contact, they would get the index questionnaire.

The index questionnaire mainly focussed on the participant’s most recent scabies episode, starting with questions about diagnosis, symptoms and treatment. We then asked about the number and type of contacts to whom the index could have transmitted the disease, the extent to which the participant notified these contacts and reasons for incomplete contact notification. Next, we provided the respondent with information about the contact-definition as used in the Netherlands for determining treatment groups and asked whether the index had notified all contacts according to this definition. We paraphrased the guideline definition [12] as follows: ‘People with whom you have had prolonged (> 15 min) or frequent skin contact since 1–2 weeks after becoming infected. This often comes down to family contacts, housemates, friends and guests staying overnight. This also applies if you did not have any symptoms of scabies during that period, such as itching, rash, etc. Examples of skin contact are holding hands, sitting next to each other, or sharing a bed (bed partners, lovers, guests staying overnight). Via intensive use of each other’s clothing, towels and bedding or, for example, a shared sofa in the communal living room, the scabies mite can also be transmitted.’

The contact questionnaire focused on the most recent contact notification event, with questions about relationship to the notifier, actions taken after receiving the notification (e.g. including simultaneous treatment, hygienic measures such as washing clothes and bedding [12]), compliance with recommended measures and reasons for incomplete compliance.

Both questionnaires also contained questions about the respondent's health care seeking behaviour, including questions on which healthcare providers were contacted. After completing all relevant questions, respondents were asked if they wanted to participate in the raffle for the €50-gift voucher. The Survalyzer application was used to develop and publish the questionnaire and to collect responses. This application assures confidential use of personal data according to EU General Data Protection Regulation (GDPR). We used closed questions with pre-programmed answer options where possible.

The questionnaire design was developed by the study team, on the basis of medical doctors’ experience with treating students with scabies as well as the researchers’ experience with similar studies on health-related preventive behaviours, including on contact notification [16, 17]. Variables were informed by Dutch scabies guidelines [12]. Questions were drafted on the basis of the study objectives and pre-tested with a small sample of members of the target population, to assess clarity and time needed for completion of the questionnaire. No formal validation was performed. The questionnaire also contained questions on psychosocial determinants of notification and treatment behaviours, but their analysis is beyond the scope of this study.

### Data cleaning and statistical methods

Data were analysed in SPSS version 28. We excluded respondents from analysis, who did not meet the inclusion criteria (age outside age range, not studying in a higher education institution, no scabies experience < 1 year prior to data collection). To our surprise, a high number of participants did not enter their age. We cannot explain or prove why this happened, but we belief it may have been a technical issue. Considering the consistency in the remaining data that those participants entered (e.g. details on their living conditions and scabies experience), we had no doubt about their eligibility and kept them in the analyses. We also removed individuals from the analysis, if they had > 5 missing values on key variables (describing contact notification behaviour or compliance with preventive measures advised for contacts) or because of too many missing values or inconsistency in the answers. Background variables and behavioural patterns (contact notification versus compliance with measures) were studied by calculating frequency distributions and means; significance of differences was tested with chi-square tests and analysis of variance (ANOVA). Figures were created using the ggplot package of R version 4.3.1.

## Results

### General information

The introductory questionnaire was completed by 807 consenting students. After excluding respondents who did not meet the inclusion criteria or had too many missing variables, 579 respondents remained. Of them, 308 completed the index questionnaire and 269 the contact questionnaire.

Table 1 gives a description of respondent characteristics. About half of the respondents came from Rotterdam and Leiden, where we focussed our recruitment. There were slightly more females and university students (as opposed to higher vocational education student) among contacts than among indexes. Scabies experience was often quite recent: respectively, 24% and 51% of indexes had scabies at the moment of the survey and or in the 3 months preceding the survey, and 13% and 69% of contacts received the last notification in the last week and last 3 months preceding the survey. About 40% of our contacts had also experienced scabies themselves and about 70% of the indexes had ever received a contact notification for scabies.

**Table 1:** Characteristics of study participants

		Indexes (*n* = 308)	Contacts (*n* = 269)	Total (*n* = 577)	
		Count (%)	Count (%)	Count (%)	*p* value
Study city	Rotterdam	87 (28.2%)	66 (24.5%)	153 (26.5%)	0.497, chi-square
	Leiden	73 (23.7%)	78 (29.0%)	151 (26.2%)	
	Utrecht	70 (22.7%)	58 (21.6%)	128 (22.2%)	
	Other^b^	78 (25.3%)	68 (24.9%)	145 (25.1%)	
Age	Mean in years (range)	21.7 (18–27)	21.5 (18–26)	21.6 (18–27)	0.216, ANOVA
	Missing	60 (19.5%)	70 (25.8%)	130 (22.5%)	
Gender	Female	196 (63.6%)	190 (70.6%)	386 (66.9%)	0.049, Fisher’s exact test^c^
	Male	112 (36.4%)	75 (27.9%)	187 (32.4%)	
	Other/unknown	0 (0.0%)	4 (1.5%)	4 (0.7%)	
Type of education	University education	244 (79.2%)	237 (88.1%)	481 (83.4%)	0.005, Fisher’s exact test
	Higher vocational education	64 (20.8%)	32 (11.9%)	96 (16.6%)	
Living situation	Living with parents/caretakers	22 (7.1%)	11 (4.1%)	33 (5.7%)	0.120, chi-square
	Living in a student house or a student housing complex with shared facilities^d^	258 (83.8%)	243 (90.3%)	501 (86.8%)	
	Living in independent home (rented or owned) with or without partner)	26 (8.4%)	13 (4.8%)	39 (6.8%)	
	Other	2 (0.6%)	2 (0.7%)	4 (0.7%)	
Number of housemates	0	7 (2.3%)	6 (2.2%)	13 (2.3%)	0.220, chi-square
	1	29 (9.4%)	20 (7.5%)	49 (8.5%)	
	2	60 (19.5%)	42 (15.7%)	102 (17.7%)	
	3	43 (14.0%)	30 (11.2%)	73 (12.7%)	
	4	44 (14.3%)	31 (11.6%)	75 (13.0%)	
	5	25 (8.1%)	22 (8.2%)	47 (8.2%)	
	> 5	100 (32.5%)	117 (43.7%)	217 (37.7%)	
Membership of student associations	Member of both study and student association	71 (23.1%)	78 (29.0%)	149 (25.8%)	0.229, chi-square
	Member of student association	153 (49.7%)	127 (47.2%)	280 (48.5%)	
	Member of study association	44 (14.3%)	40 (14.9%)	84 (14.6%)	
	Not a member of study or student association	40 (13.0%)	24 (8.9%)	64 (11.1%)	
How many persons do you know who have had scabies during the past year?	I don’t know anyone who has had scabies	1 (0.3%)	0 (0.0%)	1 (0.2%)	0.871, chi-square
	1 person	11 (3.6%)	11 (4.1%)	22 (3.8%)	
	2–5 persons	68 (22.1%)	68 (25.3%)	136 (23.6%)	
	6–10 persons	76 (24.7%)	64 (23.8%)	140 (24.3%)	
	> 10–20 persons	73 (23.7%)	60 (22.3%)	133 (23.1%)	
	> 20 person	79 (25.6%)	66 (24.5%)	145 (25.1%)	
When was the last time you had scabies yourself?	I currently have scabies	73 (23.7%)	0 (0.0%)	73 (12.7%)	
	1–4 weeks ago	81 (26.3%)	1 (0.4%)	82 (14.2%)	
	1–3 months ago	75 (24.4%)	28 (10.4%)	103 (17.9%)	
	4–6 months ago	51 (16.6%)	25 (9.3%)	76 (13.2%)	
	7–12 months ago	28 (9.1%)	31 (11.5%)	59 (10.2%)	
	> 12 months ago	0 (0.0%)	26 (9.7%)	26 (4.5%)	
	I’ve never had scabies	0 (0.0%)	158 (58.7%)	158 (27.4%)	
When was the last time you received a contact notification for scabies?	Last week	21 (6.8%)	36 (13.4%)	57 (9.9%)	
	1–4 weeks ago	37 (12.0%)	90 (33.5%)	127 (22.0%)	
	1–3 months ago	76 (24.7%)	95 (35.3%)	171 (29.6%)	
	4–6 months ago	43 (14.0%)	32 (11.9%)	75 (13.0%)	
	7–12 months ago	25 (8.1%)	16 (5.9%)	41 (7.1%)	
	> 12 months ago	12 (3.9%)	0 (0.0%)	12 (2.1%)	
	I have never had a contact notification for scabies	94 (30.5%)	0 (0.0%)	94 (16.3%)	

^b^ Other respondents studied in Amsterdam (*n* = 40), Delft (*n* = 29, Eindhoven (*n* = 19), Enschede (*n* = 9), Groningen (*n* = 15), Maastricht (*n* = 9), Nijmegen (*n* = 4), The Hague (*n* = 7), Wageningen (*n* = 2), and other (*n* = 11)

^c^ Fisher’s Exact test, calculated ignoring the few individuals with other or unknown sex

^d^ Typically sharing kitchen, bathroom; sometimes also shared living room

### Indexes

Table 2 shows the main characteristics of the indexes (*n* = 308). Almost 60% of the indexes had experienced multiple scabies episodes, and 22% even had four or more episodes. Almost two-thirds of the indexes had not received a contact notification themselves before their last episode (i.e. the person who had infected them had not notified them about possible infestation). About half (*n* = 148) of the indexes self-diagnosed the infection. Most indexes (86%) reported to have suffered from scabies (quite a bit, much or very much), and in half of the cases, it took 3 months or more to clear the symptoms.

**Table 2:** Students with scabies: characteristics of the last scabies episode (*n* = 308)

		Count	%
How many times have you had scabies in your life so far, including the last episode?	Once	125	40.6%
	Twice	73	23.7%
	Three times	45	14.6%
	Four times or more	65	22.1%
Were you notified beforehand that you could have contracted scabies, and by whom?	Yes, by the source of my infestation	96	31.2%
	Yes, by someone else	15	4.9%
	No, I was not notified beforehand	195	63.3%
	Other (persistent or recurrent infection)	2	0.6%
Who diagnosed the scabies?	General practitioner	113	36.7%
	Dermatologist	45	14.6%
	Municipal health service (GGD)	2	0.6%
	Self-diagnosis	148	48.1%
To what extent have you suffered from scabies-related complaints such as itching and rash?	I did not suffer at all	4	1.3%
	I suffered a bit	39	12.7%
	I suffered quite a bit	71	23.1%
	I suffered much	97	31.5%
	I suffered very much	97	31.5%
How long did it take for you to be completely cured of scabies?	Less than 4 weeks	49	16.0%
	1–2 months	106	34.4%
	3 months or more	152	49.4%
	Missing	1	0.3%
Approximately, how many contacts did/do you have, approximately, whom you might have infected with scabies?	0	19	6.2%
	1–5	185	60.1%
	6–10	60	19.5%
	More than 10	44	14.3%
Type of contacts, who may have been exposed to the infection by the index case (percentage of all indexes, including those with 0 contacts, *n* = 308)	(Bed) partner(s)	241	78.2%
	Household members, other than family	213	69.1%
	Friends, who are not household members	131	42.5%
	Family members	117	37.9%
	Acquaintances from student association	62	20.1%
	People visiting my household members or family	30	9.7%
	Acquaintances from study association	13	4.2%
	Acquaintances from sport club	5	1.6%
	Other	1	0.3%
Have you notified these contacts (possibly anonymously) about possible infection with scabies? (percentage of indexes, reporting to have had > 1 contact, *n* = 289)	No, I did not notify any of my contacts	10	3.5%
	Yes, I notified some of my contacts	52	18.0%
	Yes, I notified about half of my contacts	16	5.5%
	Yes, I notified most of my contacts	97	33.6%
	Yes, I notified all my contacts	114	39.4%
According to the presented guideline definition of contacts, have you notified all your possible contacts? (percentage of all indexes, *n* = 308) ^a^	According to this definition, I notified: Not any of my contacts	9	2.9%
	Some of my contacts	35	11.4%
	About half of my contacts	20	6.5%
	Most of my contacts	93	30.3%
	All of my contacts	150	48.9%
	Missing	1	0.3%
Which health care providers did you consult about your scabies infestation? (multiple answers possible)	Did not contact any health care provider	32	10.4%
	Contacted the following health care provider		
	-General practitioner	274	89.0%
	-Dermatologist	70	22.7%
	-Municipal Health Service (GGD)	44	14.3%
	-Any of the above	276	89.6%

^a^ See methods section for the guideline definition

**Fig. 1:**
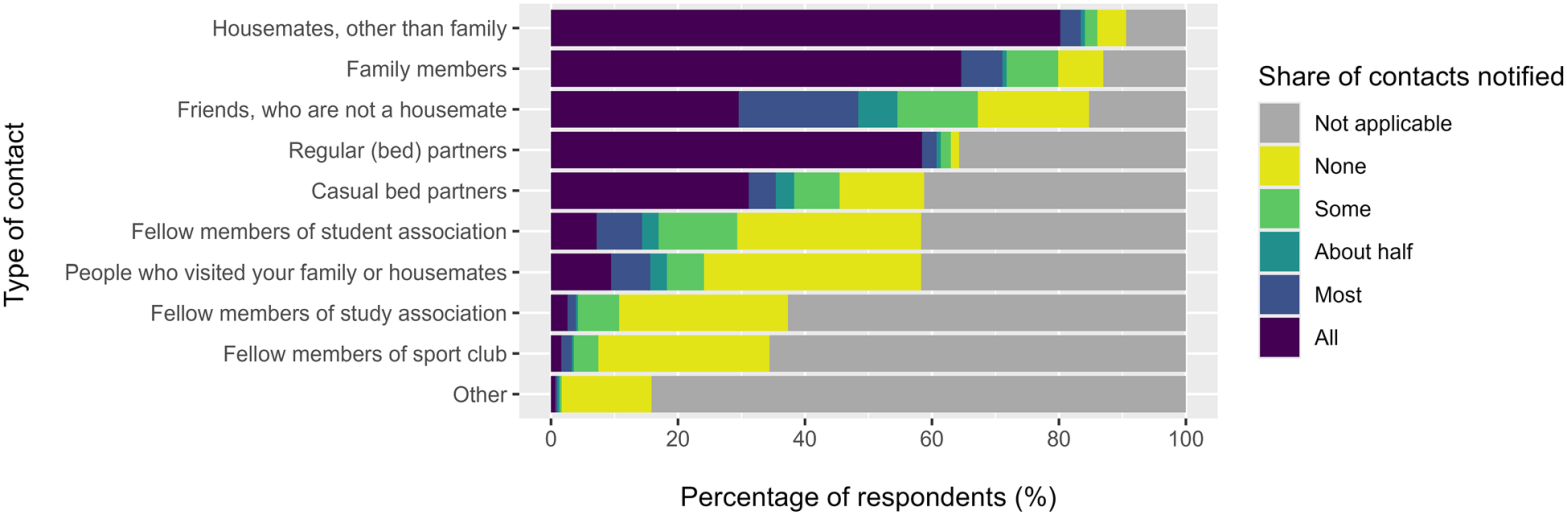
Extent to which students with scabies (indexes) notify their contacts, by type of contact. Respondents answered this question on the basis of their own definition of contacts, i.e. before being presented the contact definition from the Dutch treatment guidelines

About one-third of the indexes reported having six or more contacts, most commonly bed partners, household members, friends and family (Table 2). While 39% notified all their contacts, 21% notified none or only some. The extent to which contacts are notified varied, as shown in Fig. 1: if applicable, > 90% indexes notified all or most of their regular bedpartners and housemates, and about 74% of indexes also notified all their family members. However, only 53% of the indexes with casual bedpartners notified all of them with 23% notifying none. Friends, visitors and fellow members of study/student associations and sport clubs were notified less often. Gender subgroups showed no significant differences. The indexes who reported not to have notified all their contacts gave the following reasons for this (*n* = 175): low likelihood of having passed on the infection (*n* = 106, 61.3%), fear (*n* = 48, 27.7%), uncertainty about who should be notified (*n* = 51, 29.1%) or lack of contact information (*n* = 12, 6.9%). Other reasons mentioned in free text fields included a long diagnostic delay (contacts’ risk was deemed negligible, if they had not contracted it during the prolonged period of diagnostic uncertainty), notification being unnecessary as index avoided physical contact, or postponing notification until after completing treatment. One person indicated to have had too many (indirect) contacts, including via fomites (e.g. coat rack), making it impossible to notify everyone.

When we presented the indexes afterwards with the definition of who should be notified according to the Dutch national guidelines, the proportion of respondents indicating to have notified all their contacts increased from 39 to 49%, and the proportion of respondents indicating to have reported none or only few of their contacts declined from 21 to 14% (Table 2).

Ten percent of indexes had not consulted any health care provider for their scabies, meaning that their diagnosis was also not confirmed. Of the indexes, 89%, 23% and 14% respectively had consulted a GP, dermatologist or the municipal health service (Table 2). Respectively 39%, 14% and 32% of respondents were not or not at all satisfied with their contact with these healthcare providers. Frequently cited reasons for dissatisfaction were no or late diagnosis, or receiving conflicting, not enough or incorrect information/advice.

### Contacts

Table 3 shows the main characteristics of the contacts (*n* = 269). Nearly half (47%) of contacts had received three or more notifications in the past year and 10% even six or more. Most notifications (86%) came directly from the person with scabies. The notifier was often a housemate (57%), friend (27%) or someone from a study or student association (17%), and less often a regular or casual bedpartner (13% or 10%). Three-quarters of contacts had no scabies-related complaints when notified. Two-thirds of the contacts sought advice, usually from the internet, people who have had scabies, or other social contacts; fewer consulted a GP or dermatologist.

**Table 3:** Students who were notified for scabies: characteristics of the latest notification and measures taken (*n* = 269)

Question	Answer category	Count	%
How often have you been notified in the past 12 months about possible infestation with scabies?	1 or 2 times	142	52.8%
	3–5 times	100	37.2%
	6 + times	27	10.0%
By whom were you notified?	By the potential source	230	85.5%
	By someone else	39	14.5%
What was your relationship to the person who notified you? (multiple answers were possible)	A housemate	154	57.2%
	A family member	8	3.1%
	A friend	73	27.1%
	A casual bedpartner	28	10.4%
	A regular bedpartner	35	13.0%
	Someone from the study or student association	47	17.5%
	Other	8	3.0%
Did you have scabies-related complaints, such as itching and rash, when you received the notification?	No complaints	198	73.6%
	Some complaints	52	19.4%
	A moderate amount of complaints	7	2.6%
	Many complaints	6	2.2%
	Very many complaints	6	2.2%
Who did you ask for advice? (multiple answers possible, number and percentage indicating to have asked the given source for advice)	The person who notified me about the potential infestation with scabies	74	27.5%
	General practitioner	69	25.7%
	Dermatologist	12	4.5%
	Someone who has had scabies	106	39.4%
	Guideline website of the Dutch Institute for Public Health and the Environment	99	36.8%
	One of my social contacts	103	38.3%
	Other	4	1.5%
	Did not seek advice	90	33.5%
Looking back on how you acted after you received a contact notification: did you take the right measures according to the guidelines from the RIVM?	No, I have not followed any of these measures	29	10.8%
	Yes, I have partially followed these measures	22	8.2%
	Yes, I have largely followed these measures	43	16.0%
	Yes, I have fully/exactly followed these measures	175	65.1%
Which of the following healthcare providers or health authorities have you contacted (by phone, directly, or by email) regarding your scabies contact alert? (multiple answers possible)	Did not contact any of the healthcare providers below	134	49.8%
	Contacted the following healthcare provider		
	-General practitioner	125	46.5%
	-Dermatologist	24	8.9%
	-Municipal Health Service (GGD)	24	8.9%
	-Any of the above	135	50.2%

**Fig. 2:**
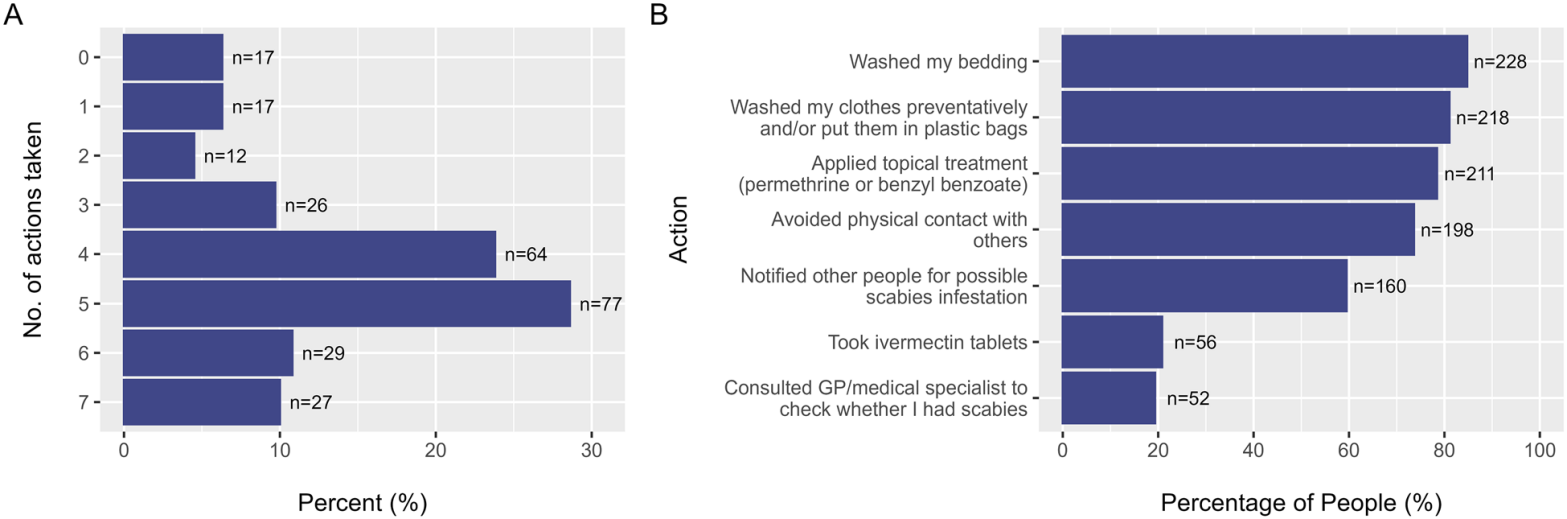
Contacts’ responses after receiving their latest contact notification (*n* = 269). **A:** number of actions taken by contacts. **B:** Percentage of respondents reporting having taken the given action

Figure 2 gives information about the number and type of actions taken by contacts, after receiving a contact notification. Some students did not take any measures after notification (*n* = 17, 6%), while a large portion of students appropriately combined treatment (permethrin or ivermectin) with washing of clothes and bedding (*n* = 208, 77%). Many also avoided physical contact with others, and for some (*n* = 12) this was the only action taken. A large part of the contacts (*n* = 160, 59%) notified others about their possible scabies infestation. Other measures taken included avoiding sitting on upholstered sofas and chairs, washing (or put in sealed bags) their pillows and stuffed animals and cleaning their rooms.

When we presented the contacts afterwards with an overview of recommended measures for contacts according to the guidelines of the Dutch Institute for Public Health and the Environment (RIVM), 65% of students indicated to have applied all recommended measures, and 11% indicated that they did not apply any of the recommended measures (Table 3). Main reasons for not or only partially applying the recommended measures were low perceived risk of infestation (63%) and absence of scabies-related symptoms (51%). Other reasons included high costs (23%), being unfamiliar with the guidelines or finding them unclear (17%), too much work (14%) and unavailability of medication (5.6%).

Half of the contacts (*n* = 125, 47%) reported to have contacted a GP, dermatologists and/or municipal health service (Table 3). While satisfaction with healthcare provider contacts was slightly higher than for indexes, the reasons for dissatisfaction were similar.

## Discussion

A digital survey was used to collect information on the extent to which students with scabies notify all their contacts and the extent to which contacts take adequate measures. In addition, their experiences with scabies and healthcare providers were evaluated. We found that contact notification by indexes and subsequent actions taken by contacts are not always fully in line with Dutch recommendations, which may hamper effective control. Our study further shows that most respondents knew multiple peers who had had scabies. Many indexes reported having suffered multiple scabies episodes, while many contacts had received multiple contact notifications and also had suffered from scabies themselves. The information received from healthcare providers was often not rated very positive.

About 20% of indexes reported to have notified only half or even fewer of the contacts recommended by Dutch guidelines. This may not be too problematic, if at least all high-risk contacts are notified. However, while most indexes notified all their housemates and regular sexual partners (all at high risk), casual bedpartners (also at high risk) were often not notified. Failure to notify all bedpartners is a known issue for sexually transmitted diseases [18]. It is associated with a risk of onward transmission [19], especially with one-off partners who tend to have larger, non-exclusive sexual networks [20]. For other less-notified contact types (e.g., members of a study or student association, members of a sport club) the intensity of contact and associated risk of contracting scabies from the index will often be low. Notifying them will then not be required according to the guidelines.

Two-thirds of the indexes were not notified before their latest scabies episode, which seems at odds with the relatively high contact notification rates reported by indexes, at least for high-risk groups. Various factors could explain this discrepancy. Firstly, indexes may have overestimated their contact notification rate. If they had been aware that they should notify all their contacts since 4–6 weeks before the onset of symptoms, they might have estimated lower rates. It is also possible that they have forgotten about some contacts in this period, especially if there was also a diagnostic delay. This 4–6-week period was not explicitly mentioned in the questionnaire, but could have been helpful for respondents to identify all their contacts. We recommend including such information in future studies on index notification behaviour. Secondly, unnotified indexes may have belonged to less frequently notified contact groups that can play a considerable role in transmission (e.g. casual bedpartners). Thirdly, transmission may occur indirectly via fomites (e.g. bedding, clothing, upholstered furniture) [21], which could potentially blur the link between source and contact and make contact notification difficult or inapplicable. Students belief strongly in these indirect transmission routes [22], although it is deemed rather uncommon in ordinary scabies [1]. Finally, owing to self-selection our respondent group may be biased towards students who are more motivated to follow treatment steps and notify contacts, with less interested students potentially less likely to participate or notify.

About 80% of notified contacts took preventive measures, including applying scabicidal cream (permethrin), washing bedding and washing clothes or sealing them in plastic bags for at least 3 days. Many also avoided physical contact with others, which was recently added to the national guideline. In addition, some contacts also informed others about their possible infestation. Notification is only recommended if a contact has scabies-related complaints [12], as premature notification might cause unnecessary concern and could potentially lead to overtreatment and treatment-fatigue. The willingness to take extra precautions illustrates the students’ strong motivation to prevent infestation and onward transmission. Our data were not detailed enough to assess whether the response was appropriate given the intensity of the contact with the initial index case.

Some students delay taking the recommended measures (treatment, hygiene) and instead adopt a ‘watchful waiting’ strategy, acting only if symptoms appear. This approach may be appropriate for contacts with minimal exposure to the index case, especially since many students felt their risk of scabies was low. However, few students remarked that scabies occurs so frequently in their house or social network, that applying all measures at every notification is not practical. In such situations, mapping the broader transmission network and considering group treatment may be more efficient.

Contact tracing can be an effective public health tool for controlling infectious diseases [23]. It is also important for scabies, as untreated contacts can become cases themselves and can reinfect the original index case [7] or spread the disease further to others. The Dutch scabies treatment guidelines recommend treating all household members and other close contacts [12]. This guideline may not be directly applicable to student houses with many housemates, where the transmission risk depends on the extent to which spaces are shared (e.g., living room, kitchen, bathroom, toilet or coat rack). This is not accounted for in national guidelines, forcing students to make their own judgement.

Our study has several limitations. Firstly, we specifically asked students with scabies experience to respond. This entails a risk of selection bias, with students who take the scabies problem more seriously being more likely to respond. We aimed to minimize this effect by advertising the study widely among all students and raffling gift vouchers among respondents as incentive. Secondly, many indexes self-diagnosed their condition and in at least 10% of cases (namely those not seeing a health worker) the diagnosis was not confirmed. This can be problematic, as scabies is easily confused with other dermatological conditions owing to shared signs and symptoms. The correctness of diagnosis is not that relevant when assessing whether indexes take correct action when they think they have scabies. However, incorrect diagnosis can introduce a bias in other outcomes, such as the number of scabies episodes experienced by respondents, the amount of suffering experienced, the required treatment duration, or satisfaction with healthcare providers. Thirdly, our study provides limited insight into whether the behaviour was adequate and timely, as we lack information on the type, timing and intensity of contact between indexes and their contacts and between contacts and the alleged source of their infection. For example, the validity of reported contact notification rates by indexes is uncertain, as we do not know whether indexes correctly identified all their potential contacts (including any contacts during the presymptomatic period). Also, when a contact received a notification from a housemate (one of many) with whom he/she has had little contact and has not shared facilities, a watchful waiting approach may sometimes be acceptable, provided that secondary contact (e.g. via housemates who are in closer contact with the index) or contact through fomites is also limited. Fourthly, our questionnaire relied on self-reported behaviours about scabies experiences from up to 1 year ago, making the data susceptible to recall and possibly social desirability bias. In addition, for students with scabies experience both as index and as contact, it may have been difficult to always maintain the focus on the last event. Indeed, in open questions we sometimes noticed that contacts had answered a question from the perspective of an index. Valuable additional information will come from in-depth interviews, which were held with 15 indexes [22] and 15 contacts.

Despite the limitations, our study clearly shows that contact notification among students is not optimal, with many never-notified indexes and both over- and under-notification of contacts. To improve the notification behaviour and subsequent actions by contacts, indexes need better information or support to identify contacts who should be notified and contacts need better information on what measures should be taken depending on the type and intensity of contact. This information should consider the specific living conditions of Dutch students and the transmission risk associated with direct and indirect contact. Extra effort may be needed to motivate students to always notify all contacts, including casual bedpartners. An analysis of psychosocial-determinants of notification-behaviours may provide important additional information to help design interventions to improve these behaviours.

Besides improved contact notification, individual-level disease management needs strengthening. About 90% of the indexes and half of the contacts had consulted a healthcare provider, usually their GP, but many were dissatisfied with scabies-related consultations due to late diagnoses and insufficient or incorrect information. There is room for improvement, e.g. by training GPs in recognition of key symptoms and assisting patients with contact identification. Better information on the many steps involved in treatment is also important, and leaflets translating scabies treatment guidelines [12] in clear step-by-step treatment instructions for ivermectin and permethrin have already been developed. There is a need for simpler effective treatment that avoid the difficulties of topical treatment, repeated doses and fomite control. Moxidectin is an interesting candidate due to its long half-life time [24] and trials are ongoing to assess its effectiveness. Offering free or low-cost medication may also help to improve treatment compliance among contacts, who frequently mentioned costs as a reason for non-adherence to guidelines.

It is uncertain to what extent improvements of notification behaviour and of measures taken by contacts will help to stop the ongoing epidemic among students. If the scabies incidence remains high, a screen-and-treat approach could be considered, involving screening of the whole target population for clinical manifestations of scabies and treating all detected cases with their contacts [25]. However, this is time-consuming and expensive and asymptomatic carriers would be missed and not treated. Mass drug administration (i.e. offering treatment to all members of a high-risk target population, without individual diagnosis) could be considered as an alternative, but is only recommended if the scabies prevalence is 10% or more [25].

## Conclusions

This study reveals deficiencies in contact notification among Dutch students affected by scabies. Most indexes were not notified by a source person. Less than half of the indexes indicated to have notified all their contacts, with serious under-notification of casual sexual partners and a significant proportion notifying none or only some of their contacts. Notified contacts often take the recommended measures, although some prefer a watchful waiting strategy. Some contacts prematurely notify their own contacts. Our research provides guidance for education and health policy to improve contact notification and preventive treatment.

## Data Availability

Data supporting the main conclusions of this study are included in the manuscript.
